# Influence of stimulus material and effector specificity on performance in the measurement of interference control in adolescent handball and soccer players compared to non-player controls

**DOI:** 10.1007/s00426-026-02327-0

**Published:** 2026-06-15

**Authors:** Florian Heilmann, Leif E. Langsdorf, Torsten Schubert

**Affiliations:** 1 Movement Science Lab, Institute for Sport Science, Martin Luther University Halle-Wittenberg, Halle (Saale), Germany; 2 Institute for Psychology, Martin Luther University Halle-Wittenberg, Halle (Saale), Germany

**Keywords:** Executive functions, Interference control, Effector specificity, Ecological validity, Adolescent sports players

## Abstract

This study investigated how stimulus material and effector specificity influence interference control (a form of inhibitory control) in adolescent players. Ninety-one males aged 15–18 years (28 handball players, 34 soccer players, 29 non-player controls) completed nine modified Flanker task versions varying in stimulus type (abstract, handball-specific, soccer-specific) and response modality (finger, hand, foot). Mixed-design ANOVAs revealed significant main effects of congruency, stimulus type, and response modality. Responses were slower, and interference effects were larger in incongruent than in congruent trials. In addition, sports-specific stimuli and more motorically demanding response modalities were associated with increased response times compared to abstract arrow stimuli and simple finger responses. A congruency × stimulus interaction revealed reduced flanker effects for sports-specific stimuli, indicating that domain-specificity shapes interference processing. However, no consistent group differences were found, suggesting adolescent players do not exhibit broad cognitive advantages, or such advantages are context-dependent. Overall, the findings suggest that performance was primarily shaped by perceptual and motor requirements of the task, i.e., bystimulus complexity and response modality rather than by differences between athlete and control groups. Longitudinal research is needed to clarify how training and development interact to shape cognitive–motor control.

## Introduction

In many high-paced team sports, successful performance depends on the ability to rapidly interpret and respond to a stream of complex, often conflicting, sensory information while executing sports-specific motor actions under time pressure (Kibele, 2006, Xiong & Song, 2024). These demands require the application of open-motor skills, characterized by the execution of goal-directed actions in a dynamic, unpredictable environment (Gu et al., 2019, Heilmann et al., 2022). Beyond physical performance factors such as agility, endurance, and strength, cognitive domains in general, and executive functions (EF) in particular play a decisive role in enabling athletes to select appropriate responses, suppress inappropriate ones, and adapt to ever-changing in-game conditions (Cao et al., 2024, Haugan et al., 2025, Heilmann et al., 2022).

Response inhibition, a core component of EF, refers to the capacity to withhold or override a prepotent or ongoing motor response (Diamond, 2013). The ability to selectively attend to relevant information and to deal with conflicting information, such as deceptive cues, is critical in a sports context. (e.g., Güldenpenning et al., 2017, Weigelt et al., 2020). The Flanker paradigm (Eriksen & Eriksen, 1974) is a well-established paradigm to investigate selective attention and interference control by presenting target stimuli flanked by congruent or incongruent distractors. Executive functions encompass several partially separable control processes, including response inhibition and interference control (Diamond, 2013). Response inhibition typically refers to the suppression or cancellation of a prepotent or ongoing motor action, as measured, for example, with Go/No-Go or Stop-Signal tasks. In contrast, interference control describes the ability to resolve competition between simultaneously activated stimulus–response representations, such as when distracting information activates an incongruent response tendency in the Flanker paradigm. Although interference control often involves inhibitory mechanisms, such as the suppression of task-irrelevant response activation both constructs are not identical. In the present study, we therefore use the term interference control when referring specifically to performance in the Flanker task, while acknowledging that inhibitory processes contribute to this form of control. Importantly, stimulus specificity (the degree to which stimuli match an athlete’s familiar perceptual domain) and effector specificity (the correspondence between task responses and sports-trained effectors) influence interference control performance (Abernethy et al., 2001, Mann et al., 2007).

One limitation of conventional Flanker paradigms in the sports context is their limited ecological validity. Standard versions often use simple, non-sports-related stimuli (e.g., arrows or letters) and require minimal motor output, such as pressing a single key. In contrast, interference control in sports settings must be accomplished under conditions involving complex, dynamic visual stimuli and sports-specific motor responses (Hülsdünker et al., 2023). Importantly, the present study operationalizes sports relevance primarily through domain-specific visual content rather than through dynamic action information. Real sport situations are inherently dynamic and unfold over time, requiring continuous perception–action coupling and prediction of opponents’ movements. Static images allow strong experimental control and systematic manipulation of stimulus and response factors, but they represent only a simplified approximation of real-world visuomotor processing. Thus, the term sports-relevant stimulus material in the present study refers to increased domain specificity compared to abstract symbols, rather than full ecological realism. One may increase the complexity of the stimulus set and the response requirements by applying sports-relevant visual scenes rather than abstract symbols. This can also be achieved by requiring responses with both the dominant and the non-dominant effectors. Both manipulations have been shown to prolong reaction time (RT) in interference tasks, likely due to the increased perceptual and cognitive demands (Büchel et al., 2022, Buschman & Kastner, 2015, Scharfen et al., 2022). These modulations directly influence task performance measures such as flanker interference effects (Chanceaux et al., 2014).

Building on this, the concept of effector specificity in interference control has recently been highlighted by Heppe & Zentgraf (2019), who demonstrated sports-related differences in inhibitory control between hand and foot responses. Their findings suggested that long-term sports-specific training leads to effector-dominant advantages, with handball players showing superior interference control for manual responses. While Heppe & Zentgraf (2019) demonstrated effector-related differences in response inhibition (e.g., interference control) using abstract arrow stimuli, their findings do not address stimulus specificity and were interpreted as reflecting partly shared control mechanisms across effectors; the present study, therefore, extends this approach by additionally manipulating sports-relevant stimulus material alongside response effectors. Recent reviews (Albaladejo-García et al., 2023, Simonet et al., 2023) have emphasized the need for modified inhibitory/interference control protocols that incorporate sports-specific perceptual and motor demands to advance our understanding of executive functions in athletic populations. An additional important consideration concerns the developmental stage of the participants. Executive functions, and inhibitory control in particular, continue to mature throughout adolescence and into young adulthood, reflecting ongoing structural and functional development of frontoparietal control networks (Casey et al., 2005, Diamond, 2013). Investigating adolescent athletes, therefore, provides a valuable opportunity to examine whether sport-specific training is already associated with differences in cognitive–motor control while these functions are still developing. Adolescence may represent a sensitive period in which maturational processes and training-related experiences interact, allowing insights into whether potential expertise-related adaptations emerge early or only become evident after full cognitive maturation.

The current study directly addresses this research gap by implementing a modified Flanker paradigm that systematically varies both the stimulus domain (sports-relevant vs. abstract) and the response effector (hand vs. foot). By applying this protocol to handball players, soccer players, and a non-athlete control group, the study is designed to test whether aligning task demands with athletes’ sports-specific perceptual and motor expertise more accurately reflects cognitive–motor adaptations. Assessing both sports-specific and cross-domain transfer effects on interference control, therefore, provides a novel opportunity to extend the findings of recent reviews(Albaladejo-García et al., 2023, Simonet et al., 2020) and to generate deeper insights into the interplay between perceptual–motor expertise and executive functions in sports.

### Transfer question

Based on these prior studies, we hypothesized that handball and soccer players would demonstrate superior interference control compared to non-athletes, with the most substantial advantages emerging when task demands align with their sports-specific perceptual and motor expertise. More specifically, athletes are expected to show reduced flanker interference when confronted with sports-relevant stimuli rather than abstract symbols, reflecting enhanced processing efficiency within familiar perceptual domains (Abernethy et al., 2001, Mann et al., 2007). Moreover, they are predicted to exhibit better inhibitory control when responding with their trained effector (hand for handball, foot for soccer) relative to their untrained effector, consistent with sports-related adaptations in motor control (Heppe & Zentgraf, 2019). Finally, while some cross-domain transfer of expertise is anticipated, the greatest performance benefits are expected when both the stimulus domain and the effector align with the athletes’ primary sports, in line with recent reviews emphasizing the need for sports-specific interference control protocols (Albaladejo-García et al., 2023, Simonet et al., 2023).

### Methodological question

We were furthermore interested in whether more naturalistic sports-related stimulus material would show interference-related effects, i.e., longer reaction times and/or increased error rates in incongruent compared to congruent conditions. Such a result pattern is commonly reported when applying abstract stimulus material such as arrows. If that were the case, this would indicate similar interference-related processes for these different classes of stimuli (i.e., sports-related and abstract-symbolic stimuli). As a result, this would imply a validation of the common approach to use abstract stimuli for investigating interference processing, and would provide support for the assumption that, in the context of real-world sports situations, indeed, valid inferences could be drawn with both types of stimuli about the mechanisms of interference control.

## Methods

### Participants

Ninety-one male participants took part in the study, including 28 handball players, 34 soccer players, and 29 non-athlete controls, aged 15 to 18 years (M = 16.57, SD = 1.61). Athletes were recruited from competitive youth teams participating in the highest or second-highest regional leagues within their respective age categories and engaged in structured training several times per week in addition to regular competition. Inclusion criteria for the athlete groups required continuous participation in organized handball or soccer training for multiple years and active competition at the time of testing. Control participants were recruited from local schools and were required not to participate in structured competitive team sports comparable to handball or soccer training. Recreational physical activity was permitted, but no participant in the control group had a formal training history in either target sport.

Descriptive statistics for age, training, and experience across groups are presented in Table 1. ANOVA revealed significant group differences in age, *F*(2, 88) = 30.03, *p* <.001, η² = 0.41, with handball players being oldest (M = 17.86, SD = 0.85), followed by soccer players (M = 16.62, SD = 1.65), and controls (M = 15.28, SD = 1.03). T-tests with pooled SD (Bonferroni method) reveal significant differences between all groups (*p* <.01). Likewise, training experience differed significantly, *F*(2, 88) = 23.74, *p* <.001, η² = 0.38, with handball players reporting the longest practice history (M = 12.68 years, SD = 1.77), compared to soccer players (M = 7.25 years, SD = 3.99) and controls (M = 8.98 years, SD = 2.79, no history in handball or soccer) with significant differences between all groups (*p* <.01). In summary, significant group differences were found for age and training experience between all groups.

**Table 1:** Means, standard deviations, 95% confidence intervals, and ANOVA results for age, training age, height, and weight across groups

Variable	Group	*n*	M	SD	95% CI+	95% CI-	ANOVA
Age	soccer	34	16.62	1.65	17.20	16.04	F(2, 88) = 30.03, *p* <.001, η² = 0.41
	handball	28	17.86	0.85	18.19	17.53	
	control	29	15.28	1.03	15.67	14.89	
Training age	soccer	34	7.25	3.99	8.94	5.56	F(2, 88) = 23.74, *p* <.001, η² = 0.38
	handball	28	12.68	1.76	13.36	11.99	
	control	29	8.98	2.79	10.04	7.92	

M = mean; SD = standard deviation; CI = confidence interval; η² = partial eta squared. Confidence intervals represent 95% CI around the mean. Significance levels: * *p* <.05, ** *p* <.01, *** *p* <.001.

An a priori power analysis was conducted using G*Power 3.1.9.4 based on the effect sizes reported by Heppe & Zentgraf (2019). To obtain a conservative minimum sample-size estimate, we modelled the simplest relevant contrast (athletes vs. controls) for a within–between interaction with two measurements (congruent vs. incongruent). Using the lower bound of the 60% confidence interval of the reported effect (converted to *f* = 0.33), assuming α = 0.05, power (1–*β*) = 0.80, and a mixed-design ANOVA, the required total sample size was *N* = 58. The present study included three groups (handball, soccer, controls) with a total sample of *N* = 91, thus exceeding this conservative minimum and providing sufficient sensitivity for the full 3 (group) × 2 (congruency) × 3 (stimulus) × 3 (response) design. The Declaration of Helsinki and the APA’s ethical guidelines were followed in the study protocol. The study was approved by the university’s ethical committee (approval number of the ethical committee of Martin-Luther-University Halle-Wittenberg: 2412HFS).

## Measurements

**Fig. 1:**
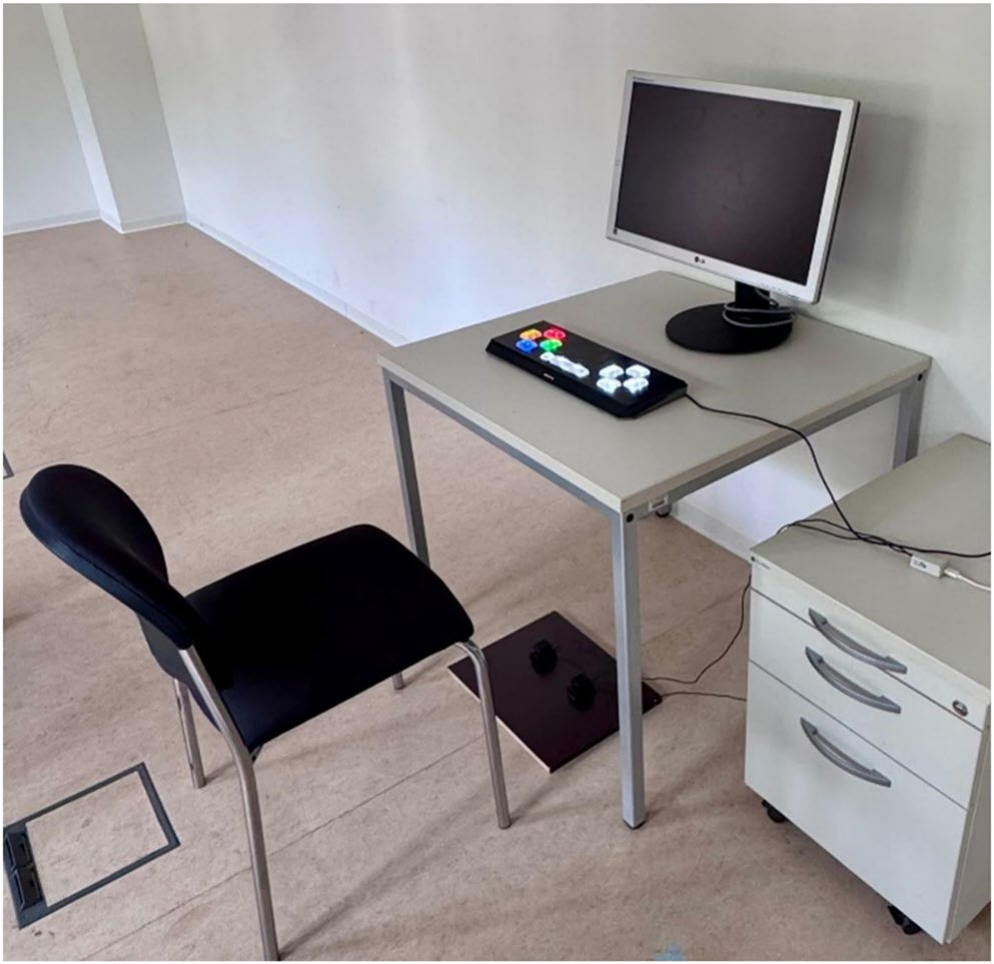
Experimental setup for the modified Flanker task. Note. Participants were seated in front of a 19-inch monitor and responded using a QWERTZ keyboard (finger responses), an external button panel (hand responses), or foot pedals (foot responses)

The Flanker tasks were examined using Inquisit Lab 6 (Millisecond Software LLC, Seattle, WA, USA) on a 19-inch screen, a QWERTZ keyboard (PC [personal computer]; see Fig. 1).

In the present study, participants completed a series of modified Flanker tasks in which five stimuli were presented in a horizontal row, with the central element serving as the target (see Fig. 1). Each trial displayed a sequence of five figures (flanker–flanker–target–flanker–flanker) centered on the screen. Depending on the condition, the figures represented either (a) abstract arrow stimuli (“rightarrow.png”, “leftarrow.png”; available in the supplementary material), (b) handball-specific player figures with one arm extended laterally to the left or right, or (c) soccer-specific player figures with one leg extended laterally to the left or right. The central element served as the target, while the adjacent elements functioned as flankers that pointed either in the same direction (congruent trials) or in the opposite direction (incongruent trials).

**Fig. 2:**
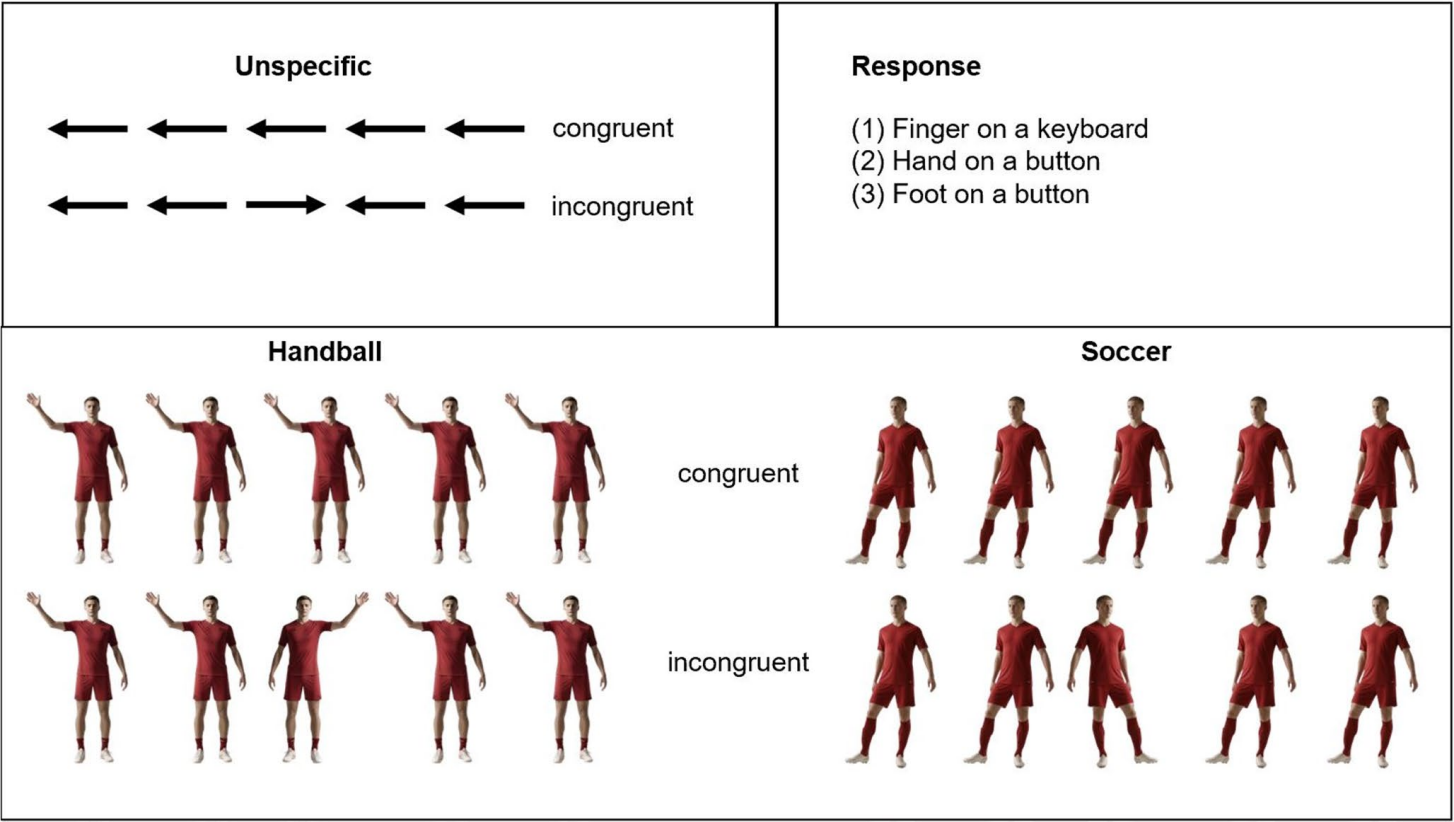
Examples of stimulus material and response modalities in the modified Flanker task. Note.Stimulus types included abstract (arrows), handball-specific (players with one arm extended), and soccer-specific (players with one leg extended) displays, each presented in congruent and incongruent conditions. Responses were given using (1) finger on a keyboard, (2) hand on a button, or (3) foot on a button

Stimuli were displayed at approximately 20% of the screen height (≈ 7–8 cm), subtending a horizontal visual angle of about 18°at a viewing distance of 60 cm. Flankers were positioned equidistantly from the target, centered at 26%, 38%, 50%, 62%, and 74% of the screen width. Each trial was preceded by a yellow fixation star (1 cm, ≈ 1° visual angle) presented for 1,000 ms. Response cues and instructions were displayed in Arial font (black text on a white background). Participants were instructed to respond to the direction of the target stimulus while ignoring the surrounding flankers. On congruent trials, all five stimuli pointed in the same direction, whereas on incongruent trials, the flankers pointed in the opposite direction to the target. Responses were made to the left when the target pointed left and to the right when the target pointed right (see Fig. 2).

The experiment consisted of nine distinct versions of the Flanker task, created by crossing two experimental factors: stimulus type and response modality. The factor stimulus type included three categories: (1) abstract arrow stimuli, (2) handball-specific player figures, and (3) soccer-specific player figures. The factor response modality comprised (1) finger responses, (2) hand responses, and (3) foot responses (see Fig. 2). Each of the nine task versions consisted of nine blocks, each containing four practice trials followed by 70 test trials. In all conditions, participants were instructed to respond to the direction of the central target stimulus while ignoring the flankers.

## Procedures

All participants or their legal representatives gave their informed consent. Participants gave the informed consent form after they had been briefed on the procedure. The participants underwent tests at their training facilities or at school (control group). The Flanker tasks were conducted in randomized order. To minimize the effects of physical exertion, the players were assessed between 10:00 a.m. and 4:00 p.m., one hour before training. The participants had to fill out a questionnaire as well, which we will not report here, but which can be found in the supplementary material. Participants’ handedness was not formally assessed, and responses were performed according to standardized task instructions independent of individual hand dominance. Consequently, handedness was not included as a factor in the statistical analyses and should be considered when interpreting effector-related results.

## Data preparation

For the Flanker task assessing interference control, several filtering steps were applied. First, all trials with incorrect responses were removed (⁓2.51% of all trials). Second, to account for extreme values, trials with response times below 200 ms or above 1,750 ms were excluded (e.g., Musculus et al., 2022; ⁓0.06% of all trials). Noparticipants were excluded due to incomplete datasets.

### Statistical analysis

A 3-group × 2 congruency × 3 stimulus × 3 response mixed ANOVA was conducted separately for response times and accuracy. Group was entered as a between-subjects factor, whereas congruency, stimulus, and response were entered as within-subjects factors. Because the participant groups differed significantly in age and because executive functions continue to develop during adolescence, we additionally conducted an age-adjusted reanalysis that included age as a covariate. This analysis examined whether the observed effects, particularly those involving group, remained stable after controlling for age-related developmental differences. Greenhouse–Geisser correction was applied where appropriate. Significant main effects and interactions were followed up using Bonferroni-corrected post hoc pairwise comparisons to control for multiple testing. No outliers exceeding 1.5 times the interquartile range were identified; therefore, none were excluded.

The factors had the following levels: group: handball, soccer, control; congruency: congruent, incongruent; stimulus: abstract, handball, soccer; response: finger, hand, foot. R and RStudio were used for all statistical analyses. The threshold for significance was fixed at *p* <.05. To resolve relevant main effects and/or interaction effects, Bonferroni-corrected post hoc simple contrasts were applied (e.g., Wilcox, 2011). The results of the age-adjusted analyses are reported alongside the original analyses to evaluate the robustness of the findings.

## Results

Tables 2 and 3 present all relevant descriptive values.

**Table 2:** Descriptive results for response times and flanker effect across groups and conditions

			Group					
			Soccer		Control		Handball	
			Mean [ms]	SD	Mean [ms]	SD	Mean [ms]	SD
Response time [ms]	abstract	finger	395.857	38.304	398.765	53.121	385.514	45.714
Congruent trials		hand	370.234	32.708	392.946	70.019	372.643	36.493
		foot	418.031	40.967	416.022	54.689	413.565	41.801
	handball	finger	400.003	43.783	407.319	52.678	388.824	39.005
		hand	380.328	33.712	400.106	72.076	380.811	41.360
		foot	435.605	53.249	427.805	59.297	424.504	46.812
	soccer	finger	405.437	39.330	425.036	66.568	401.105	39.386
		hand	392.232	45.768	410.605	68.116	385.605	39.787
		foot	438.282	48.117	443.897	60.801	446.582	49.278
Response time [ms]	abstract	finger	429.061	44.546	427.623	62.292	408.253	49.793
Incongruent trials		hand	402.816	35.887	423.261	81.266	405.788	44.130
		foot	446.279	41.299	457.782	62.915	442.706	52.878
	handball	finger	418.921	49.223	418.666	53.794	401.306	33.983
		hand	392.681	35.868	413.139	73.800	393.120	38.994
		foot	440.560	40.307	440.569	73.065	436.343	45.727
	soccer	finger	414.283	39.450	437.534	69.281	409.667	39.634
		hand	405.497	49.732	426.012	89.640	393.262	37.863
		foot	453.010	48.349	446.831	53.857	454.066	46.412
Flanker effect [ms]	abstract	finger	33.204	22.767	28.858	26.319	22.739	23.888
		hand	32.582	23.878	30.315	29.792	33.144	28.915
		foot	28.248	28.247	41.760	28.924	29.142	29.514
	handball	finger	18.918	21.788	11.347	21.788	12.482	22.937
		hand	12.353	20.113	13.033	20.885	12.308	16.265
		foot	4.955	27.670	12.765	21.870	11.839	15.978
	soccer	finger	8.847	15.996	12.497	24.788	8.563	18.621
		hand	13.264	21.980	15.406	28.430	7.658	15.332
		foot	14.727	27.256	2.933	29.980	7.484	31.088

**Table 3:** Descriptive results for accuracy across groups and conditions

			Group					
			Soccer		Control		Handball	
			Mean [%]	SD	Mean [%]	SD	Mean [%]	SD
Accuracy [%]	abstract	finger	97.76	5.35	96.86	4.54	97.24	3.6
Congruent trials		hand	99.18	2.07	97.55	3.36	97.01	4.82
		foot	96.68	5.35	96.57	4.54	96.9	3.6
	handball	finger	98.7	3.63	97.45	4.5	97.13	3.72
		hand	97.86	4.2	96.27	3.59	97.13	3
		foot	96.62	4.84	95.95	3.55	96.06	3.88
	soccer	finger	97.74	3.34	95.49	4.71	97.13	3.12
		hand	97.63	2.63	96.57	3.48	97.24	3.62
		foot	95.16	5.28	93.43	4.61	95.63	4.56
Accuracy [%]	abstract	finger	91.79	8.67	94.85	7.34	92.46	7.76
Incongruent trials		hand	93.33	5	92.66	6.15	93.53	4.49
		foot	93.99	5.14	90.63	6.11	90.3	8.3
	handball	finger	95.38	5.09	95.59	5.58	95.91	5.26
		hand	97.54	3.86	97.06	4.35	96.55	5.08
		foot	95.34	5.73	93.85	6.81	95	5
	soccer	finger	97.77	2.99	96.32	4.82	95.47	6.12
		hand	98.02	3.33	94.51	5.99	94.4	4.45
		foot	95.83	4.94	94.85	4.41	95.04	4.15

## Response time

**Fig. 3:**
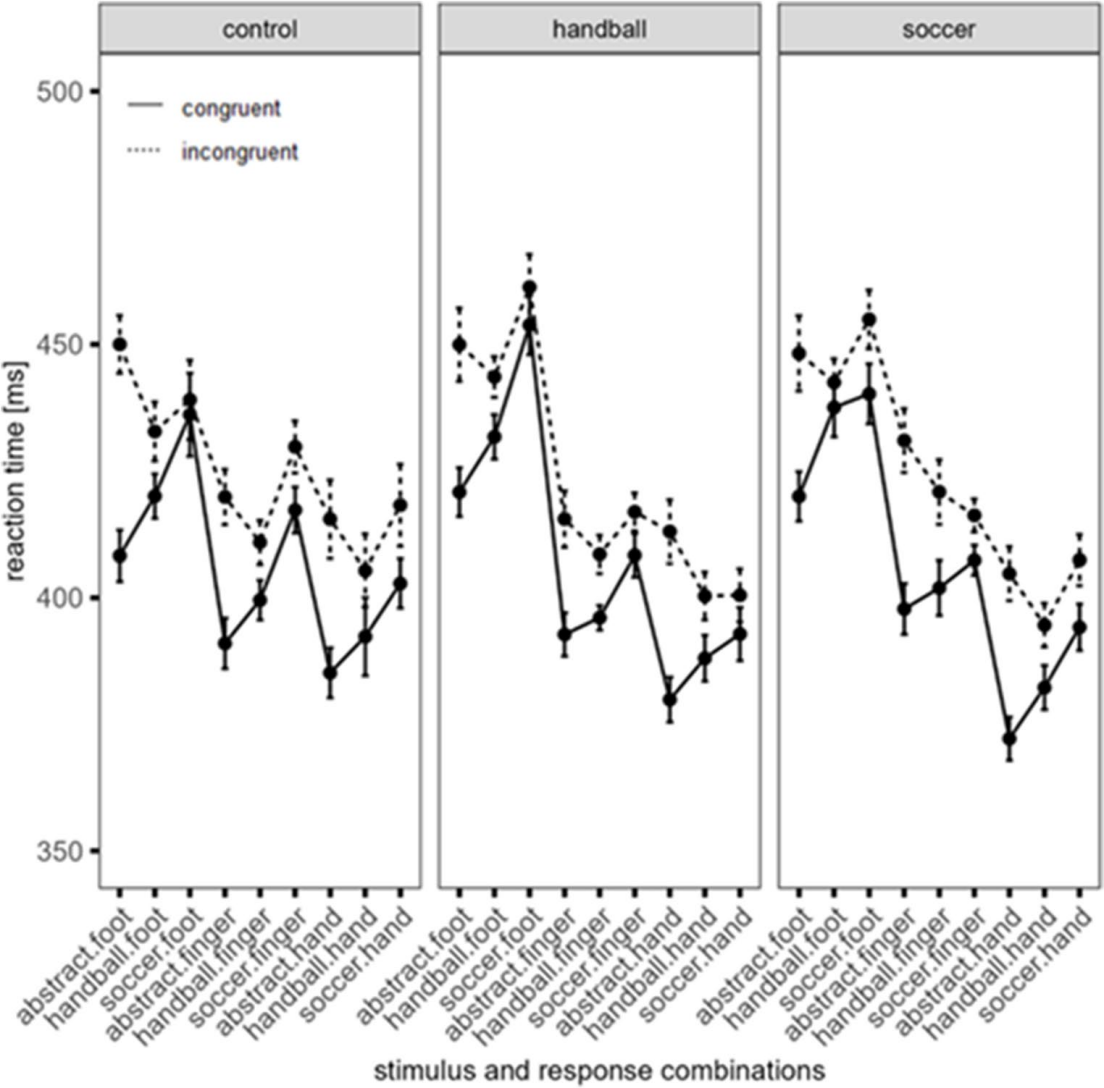
Mean reaction times (RTs) as a function of the factor group (control subjects, handball players, and soccer players), congruency (congruent, incongruent trials), stimulus type (handball, soccer, abstract stimuli), and response type (foot, finger, or hand responses). Error bars represent the standard error of the mean

Related to our research question of sports-reated modulations of interference control processes (*transfer approach*), neither the threeway interaction between the factors group, congruency, and stimulus, *F*(4, 176) = 0.45, *p* =.775, ηp² = 0.010 nor the three-way interaction between the factors group, congruency and response, *F*(4, 176) = 0.50, *p* =.735, ηp² = 0.011, reached significance. This was also not the case for the four-way interaction effect between the factors group, congruency, stimulus, and response, *F*(8, 352) = 1.62, *p* =.117, ηp² = 0.036. Hence, we found no evidence for the assumption of group-specific modulation of interference control processes (see Fig. 3).

**Fig. 4:**
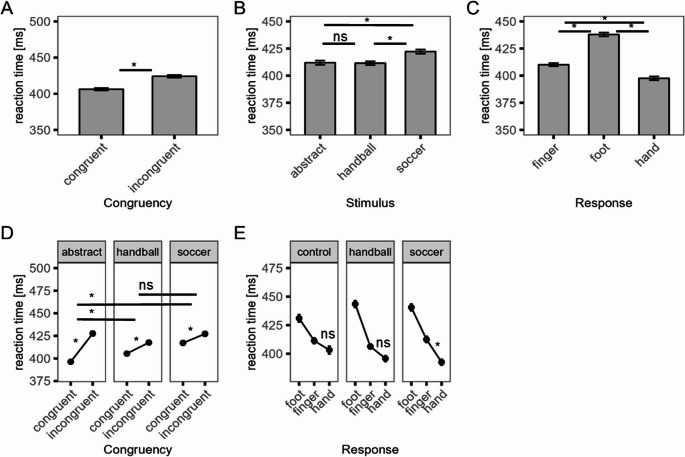
Mean reaction times (RTs) as a function of significant main effects and interactions. Error bars represent the standard error of the mean. An asterisk indicates a significant effect, and the ns indicates a non-significant effect. Black horizontal lines indicate the comparison between conditions. **A**) Shows the effect of congruency on RTs, with longer RTs in the incongruent condition compared to the congruent condition. **B**) Depicts increased RTs to soccer stimuli compared to abstract and handball stimuli. **C**) Depicts the longest RTs for foot compared to finger and hand responses. **D**) Indicates a significant congruency effect (difference between incongruent and congruent RTs) for all stimulus types. The congruency effect for abstract stimuli was increased compared to handball stimuli and soccer stimuli, while it was not different for the sport-related stimuli. **E**) Depicts that soccer players show a larger difference between finger-hand responses compared to the other groups. While all comparisons between groups were non-significant

Related to our methodological approach, we found several relevant effects, such as a large main effect of congruency, *F*(1, 88) = 253.51, *p* <.001, ηp² = 0.742, indicating slower responses on incongruent (*m* = 424 ms) than on congruent trials (*m* = 406 ms) (see Fig. 4A). This indicates that our experimental setup was effectively inducing interference effects.

The effect of the factor congruency was further specified by the interaction effect between the factors of congruency and stimulus, *F*(2, 176) = 64.10, *p* <.001, ηp² = 0.421. As can be seen in Fig. 4D, for all stimulus types, we obtained a congruency effect, all *ps* < 0.001, reflecting longer RTs in the incongruent compared to the congruent conditions. This effect was further increased in abstract stimuli, for which the congruency effect (*m* = 31 ms) was increased compared to the congruency effect with soccer stimuli (*m* = 10 ms), *t*(177.9) = 8.84, *p =*.001, *d* = 0.66, and with handball stimuli (*m* = 12 ms), *t*(172.7) = 8.31, *p =*.001, *d* = 0.63; whereas the congruency effects were comparable for soccer and handball stimuli, *t*(178.2) = − 0.97, *p =*.331, *d* = 0.07. Overall, in line with our prediction, this result pattern indicates that for sports-related stimulus material, similar interference effects emerge compared to when using abstract stimulus material. This provides further validation for the experimental approach.

We also obtained a significant main effect of the factor stimulus, *F*(2, 176) = 14.31, *p* <.001, ηp² = 0.140, reflecting longer RTs for the soccer (*m* = 422 ms) compared to the handball stimuli (*m* = 412 ms), *t*(90) = −5.58, *p =*.001, *d* = 0.59 (see Fig. 4B). Similarly, RTs to soccer stimuli were prolonged compared to abstract stimuli (*m* = 412ms), *t*(90) = 4.65, *p =*.001, *d* = 0.49. In contrast, RTs to handball and abstract stimuli (*m* = 412 ms) remained comparable, *t*(90) = − 0.18, *p =*.99, *d* = 0.02. This effect pattern probably reflects the observation that visual complexity was increased for the soccer stimuli compared to the other stimulus types, thereby prolonging information processing.

Furthermore, we obtained a significant main effect of the factor response, *F*(2, 176) = 90.09, *p* <.001, ηp² = 0.506 (see Fig. 4C). RTs were highest when participants responded with their foot (*m* = 438 ms), followed by finger responses (*m* = 410 ms), and fastest with their hands (*m* = 398 ms). In particular, foot responses were slower than finger responses, *t*(90) = −9.16, *p =*.001, *d* = 0.96, and finger responses were slower than hand responses, *t*(90) = 4.92, *p =*.001, *d* = 0.52; hand responses and foot responses also differed significantly, *t*(90) = −10.47, *p =*.001, *d* = 1.10.

Finally, we obtained a significant interaction effect between the factors group and response, *F*(4, 176) = 2.94, *p* =.022, ηp² = 0.063, reflecting larger differences between hand and finger responses in the soccer group compared to the handball and control groups (see Fig. 4E). In detail, this was mainly driven by a heterogeneous pattern of the RT differences within the different groups, as all differences across groups were non-significant, all *p´s* < 0.2. Relatedly, within the soccer group, the difference between hand (*m* = 393 ms) and finger (*m* = 413 ms) responses was especially pronounced as reflected by a significant difference, *t*(88) = 4.39, *p =*.001, *d* = 0.47. In contrast, this was not the case in the handball group (396 ms vs. 406 ms), *t*(88) = 1.97, *p =*.15, *d* = 0.21, *d* = 0.21, and neither in the control group (402 ms vs. 411 ms), *t*(88) = 2.37, *p =*.06, *d* = 0.25. These results indicate that soccer players show improved effector-related processing differences compared to the other groups.

All other effects were not significant, such as the main effect of the factor group, *F*(2, 88) = 0.92, *p* =.402, ηp² = 0.020. Consequently, athletes and their control counterparts were comparable in their processing speed; group × congruency, *F*(2, 88) = 0.56, *p* =.574, ηp² = 0.013; group × stimulus, *F*(4, 176) = 0.43, *p* =.787, ηp²= 0.010; congruency × response, *F*(2, 176) = 0.46, *p* =.635, ηp²; stimulus × response, *F*(4, 352) = 0.58, *p* =.678, ηp²; congruency × stimulus × response, *F*(4, 352) = 0.93, *p* =.449, ηp² = 0.011.

## Accuracy

**Fig. 5:**
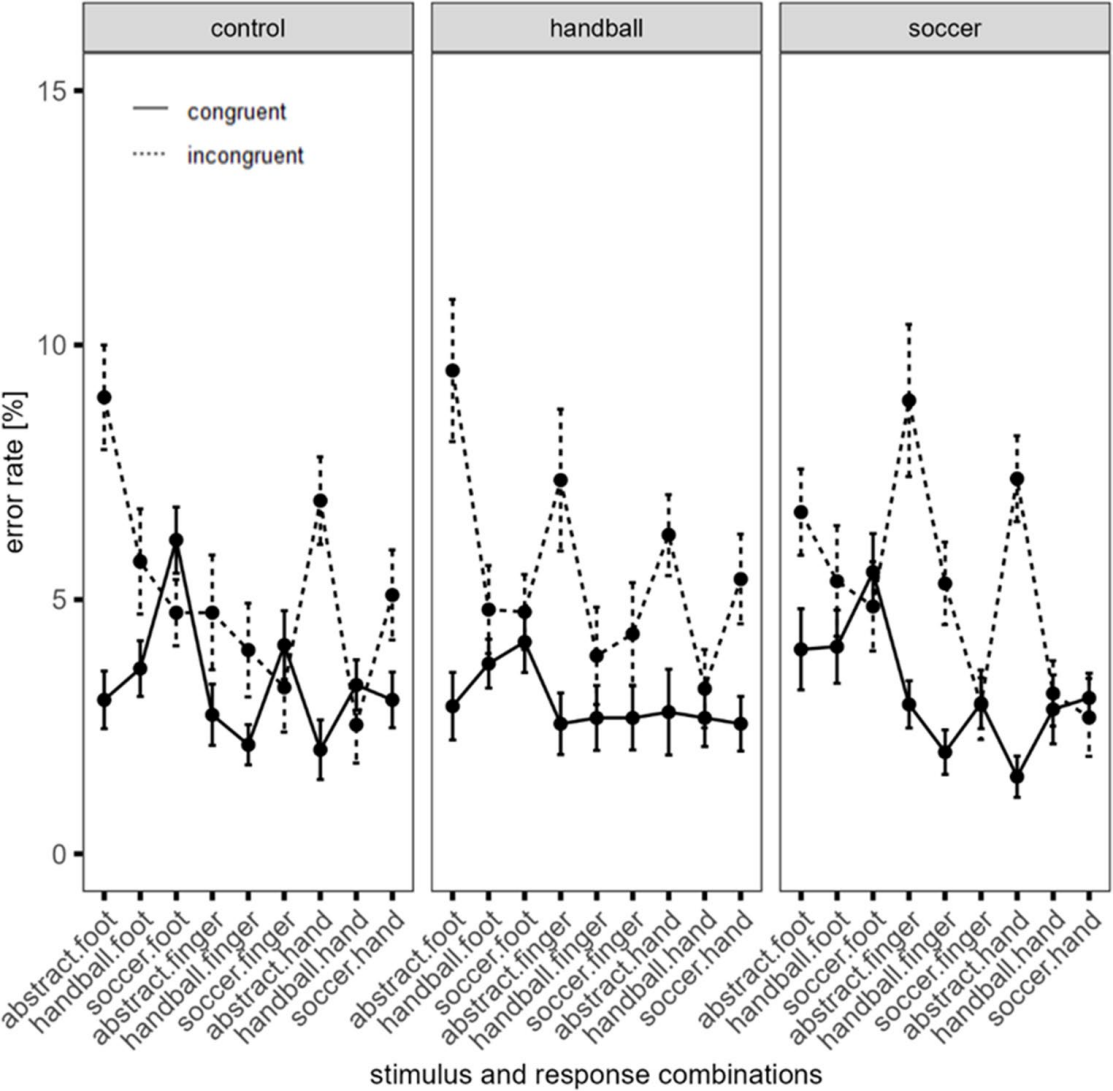
Error rates as a function of the factors group (control subjects, handball players and soccer players), congruency (congruent, incongruent trials), stimulus type (handball, soccer, abstract stimuli), and response type (foot, finger, or hand responses). Error bars represent the standard error of the mean

Related to our *transfer approach* research question of sports-related modulations of interference control processes, none of the relevant interactions reached significance. In particular, neither the three-way interaction between the factors group, congruency, and stimulus, *F*(4, 176) = 1.11, *p* =.353, ηp² = 0.025, nor the three-way interaction between the factors group, congruency, and response, *F*(4, 176) = 1.46, *p* =.215, ηp² = 0.032, reached significance (see Fig. 5). The four-way interaction between group, congruency, stimulus, and response was also not significant, *F*(8, 352) = 1.92, *p* =.56, *ηp*² = 0.042, indicating no evidence for sports-related transfer effects on interference-control performance. Overall, these results indicate no sports-related transfer effects on interference control processes.

**Fig. 6:**
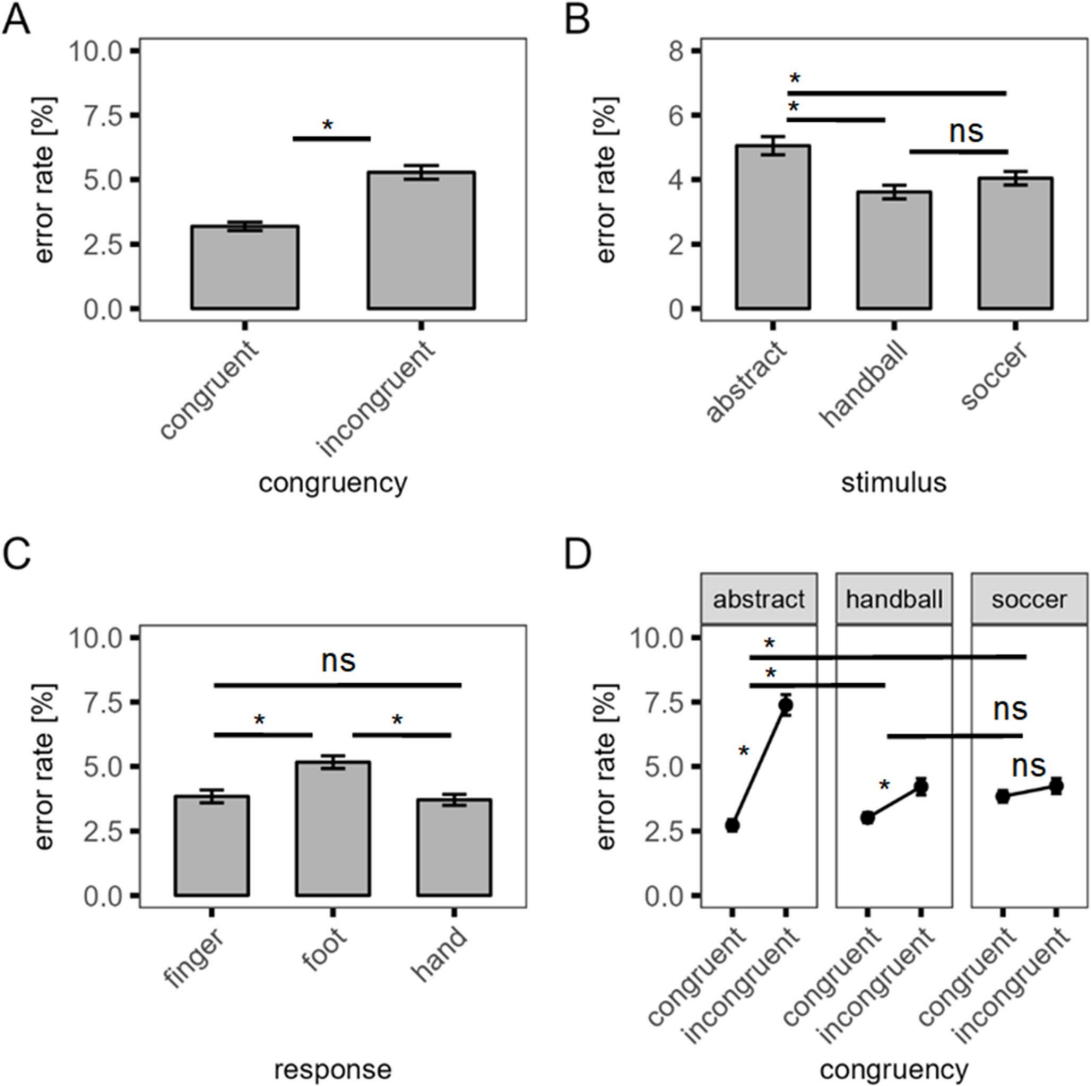
Mean error rates (%) as a function of significant main effects and interactions. Error bars represent the standard error of the mean. An asterisk indicates a significant effect, and ns indicates a non-significant effect. Black horizontal lines indicate the comparison between conditions (**A**) Shows the effect of congruency on error rates, with higher error rates in the incongruent condition compared to the congruent condition. (**B**) Depicts differences in error rates across stimulus types, with the highest error rates for abstract stimuli compared to soccer and handball stimuli. The error rates were not different between handball and soccer stimuli. (**C**) Shows error rates as a function of response type, with an increased error rate for foot responses compared to finger and hand responses. The error rates were not different between finger and hand responses. (**D**) Illustrates a significant congruency effect (difference between incongruent and congruent conditions) for abstract and handball stimuli, showing the largest congruency effect for abstract stimuli. While the congruency effect was not different between sport-related stimuli

In contrast, for our research question related to the *methodological approach*, we obtained several significant main effects and interaction effects. In detail, we obtained a strong main effect of the factor congruency, *F*(1, 88) = 67.76, *p* <.001, ηp² = 0.435, indicating that participants made more errors on incongruent (*m* = 5.3%) than congruent (*m* = 3.2%) trials (see Fig. 6A). This demonstrates a similar effect pattern as on the RTs and is in line with previous results (Heppe & Zentgraf, 2019).

Similar to the results observed for reaction times, the effect of congruency on error rates was further specified by a significant interaction between congruency and stimulus type, *F*(2, 176) = 34.55, *p* <.001, *ηp*² = 0.282, indicating that the magnitude of the congruency effect depended on the stimulus category (see Fig. 6D). More specifically, a congruency effect in error rates was observed for handball and abstract stimuli (both *p*s < 0.001), but not for soccer stimuli (*p* =.269). Post hoc comparisons revealed the largest congruency effect for abstract stimuli (m = 4.7%) compared with handball stimuli (m = 1.2%), *t*(170.2) = 5.78, *p* <.001, *d* = 0.44, and soccer stimuli (m = 0.4%), *t(*169) = 7.15, *p* <.001, *d* = 0.55, whereas the difference between handball and soccer stimuli was not significant, *t*(179.97) = − 1.54, *p* =.125, *d* = − 0.11.

We furthermore obtained a significant main effect of the factor stimulus, *F*(2, 176) = 17.21, *p* <.001, ηp² = 0.163, reflecting that participants made the fewest errors responding to the handball stimuli (*m* = 3.6%), followed by the error rate to soccer stimuli (*m* = 4%) and abstract stimuli (*m* = 5.1%) (see Fig. 6B). In detail, the error rate to handball and soccer stimuli was comparable, *t*(90) = −1.77, *p =*.238, *d* = −0.19. While the error rate was decreased for handball stimuli compared to abstract stimuli, *t*(90) = −5.268, *p <*.001, *d* = −0.55, which was also the case for soccer compared to abstract stimuli, *t*(90) = −4.007, *p <*.001, *d* = −0.42. These results indicate that sports-related naturalistic stimuli were effectively differentiated by the participants (see Fig. 6B). We also obtained a significant main effect of the factors response, *F*(2, 176) = 13.76, *p* <.001, ηp² = 0.135, reflecting the highest error rate with foot responses (*m* = 5.2%), followed by the error rate of finger responses (*m* = 3.8%), and hand responses (*m* = 3.7%) (see Fig. 6C). Comparisons demonstrated comparable error rates between finger and hand responses, *t*(90) = 0.543, *p =*.99, *d* = 0.06. In contrast, the error rates differed for finger and foot responses, *t*(90) = 3.860, *p <*.001, *d* = 0.40, as well as between hand and foot responses, *t*(90) = −4.730, *p <*.001, *d* = −0.50. These results suggest that it was more challenging for participants to respond correctly with their feet, mirroring results on the RTs, in line with previous evidence (Heppe & Zentgraf, 2019). Finally, the interaction effect between the factors congruency, stimulus, and response reached significance, *F*(4, 352) = 3.16, *p* =.014, ηp² = 0.035, indicating that congruency effects varied modestly depending on both the type of stimulus and response.

All other effects failed to reach significance: the factor group did not reach significance, *F*(2, 88) = 1.65, *p* =.197, ηp² = 0.036, reflecting similar accuracies across groups. The interaction between group and congruency failed to reach significance, *F*(2, 88) = 0.80, *p* =.452, ηp² = 0.018. Furthermore, the interaction between group and stimulus failed to reach significance, *F*(2, 88) = 1.25, *p* =.292, ηp² = 0.028. The interaction effect between group and response did not reach significance, *F*(2, 88) = 0.77, *p* =.292, ηp² = 0.017. The interaction effect between congruency and response did not reach significance, *F*(2, 88) = 0.6, *p* =.941, ηp² = 0.013. The interaction effect between stimulus and response failed to reach significance, *F*(2, 88) = 0.53, *p* =.711, ηp² = 0.012. Finally, the interaction effect between group, stimulus, and response failed to reach significance, *F*(2, 88) = 1.03, *p* =.406, ηp² = 0.023.

### Age-adjusted reanalysis

Because the groups differed significantly in age, we repeated the main analyses with age entered as a covariate. For response times, age was a significant covariate, *F*(1, 87) = 5.74, *p* =.019, ηp² = 0.062, whereas the main effect of group remained non-significant after controlling for age, *F*(2, 87) = 2.68, *p* =.075, ηp² = 0.058. Importantly, the critical group-related interactions relevant to the transfer hypothesis remained non-significant: group × congruency × stimulus, *F*(4, 174) = 1.04, *p* =.388, ηp² = 0.023; group × congruency × response, *F*(4, 174) = 0.80, *p* =.529, ηp² = 0.018; and group × congruency × stimulus × response, *F*(8, 348) = 1.55, *p* =.138, ηp² = 0.034. The main task-related effects remained significant after controlling for age, including congruency, *F*(1, 87) = 253.72, *p* <.001, ηp² = 0.745; stimulus, *F*(2, 174) = 14.74, *p* <.001, ηp² = 0.145; response, *F*(2, 174) = 90.88, *p* <.001, ηp² = 0.511; and the congruency × stimulus interaction, *F*(2, 174) = 65.46, *p* <.001, ηp² = 0.429.

A comparable pattern emerged for accuracy. Age was not a significant covariate, *F*(1, 87) = 0.13, *p* =.720, ηp² = 0.001, and the main effect of group remained non-significant, *F*(2, 87) = 1.08, *p* =.345, ηp² = 0.024. The critical group-related interactions again remained non-significant after controlling for age: group × congruency × stimulus, *F*(4, 174) = 1.03, *p* =.395, ηp² = 0.023; group × congruency × response, *F*(4, 174) = 1.40, *p* =.235, ηp² = 0.031; and group × congruency × stimulus × response, *F*(8, 348) = 1.22, *p* =.286, ηp² = 0.027. The main task-related effects also remained significant, including congruency, *F*(1, 87) = 66.98, *p* <.001, ηp² = 0.435; stimulus, *F*(2, 174) = 17.11, *p* <.001, ηp² = 0.164; response, *F*(2, 174) = 13.64, *p* <.001, ηp² = 0.136; congruency × stimulus, *F*(2, 174) = 34.58, *p* <.001, ηp² = 0.284; and congruency × stimulus × response, *F*(4, 348) = 3.19, *p* =.014, ηp² = 0.035. Taken together, the age-adjusted analyses indicate that the central conclusions were not attributable to age differences between groups: performance was primarily shaped by task-related factors, whereas no consistent sport-specific transfer effects were observed.

## Discussion

The present study examined whether adolescent handball and soccer players show sport-related advantages in interference control compared to controls, and whether sports-related stimuli elicit interference effects comparable to abstract stimuli. Based on previous research, we expected athletes to show superior interference control, particularly when stimulus material and response modality matched their sport-specific perceptual and motor experience (Abernethy et al., 2001, Mann et al., 2007, Heppe & Zentgraf, 2019). Specifically, we predicted reduced flanker interference for sports-related stimuli and enhanced performance when responding with the trained effector, namely the hand in handball players and the foot in soccer players. Because the Flanker paradigm assesses interference control arising from response competition, whereas the Stop-Signal Task primarily measures action cancellation, the present study extends previous work by targeting a related but distinct inhibitory mechanism. In addition, we examined whether sports-related stimuli produce interference effects similar to abstract stimuli, which would support the use of controlled Flanker paradigms to investigate cognitive control processes relevant to sport contexts (Albaladejo-García et al., 2023).

The present results provided only partial support for our predictions. We obtained robust interference effects, which confirmed the sensitivity of the paradigm and demonstrated influences of stimulus type and response modality; however, we did not observe the expected sport-specific advantages in athletes compared to controls. Thus, the findings suggest that task-related perceptual–motor demands influenced performance more strongly than, sport-related expertise, highlighting the importance of contextual task characteristics rather than athletic status alone.

Overall, the analyses revealed no main effect of group, indicating comparable performance across soccer players, handball players, and controls. In contrast, strong main effects of congruency, stimulus, and response confirmed that task demands substantially influenced performance, with slower responses for incongruent trials, sports-specific stimuli, and motorically demanding effectors. Significant congruency × stimulus and group × response interactions showed that interference varied across stimulus types and that response-related differences depended modestly on group, although post-hoc analyses revealed no consistent athlete-specific advantages (Hüttermann et al., 2014, Voss et al., 2010).

These findings reinforce the notion that task demands and ecological manipulations exert a much stronger influence on inhibitory performance than group membership alone. The absence of a group main effect aligns with recent reviews that question the robustness of broad, domain-general executive function advantages in athletes (Kalén et al., 2021). While earlier meta-analyses suggested athletes outperform non-athletes on inhibition tasks (Heilmann et al., 2022, Voss et al., 2010), more recent evidence, such as the present study, suggests that such effects are inconsistent and may depend heavily on the specific task context and the match between motor and perceptual demands (Botvinick et al., 2001, Ridderinkhof et al., 2004).

The robust main effects of congruency, stimulus, and response highlight the sensitivity of interference control to perceptual and motor context. Responses were slower for incongruent than for congruent trials and were influenced by stimulus type and response modality, indicating that both perceptual complexity and effector requirements shaped performance. These findings replicate and extend previous work showing that more realistic task demands increase cognitive load (Heppe & Zentgraf, 2019). From an embodied cognition perspective, response selection and conflict resolution arise from interactions between sensory input, motor representations, and task demands rather than from abstract cognitive processes alone (Barsalou, 2008). Thus, the present findings suggest that interference control is shaped by perception–action couplings. Related to the methodological validation of sports-related stimuli, interference effects were observed not only for abstract stimuli but also for sports-related stimulus material. Although abstract stimuli produced the largest interference effects in RTs and error rates, the presence of comparable effects across stimulus types suggests that similar cognitive control and conflict-monitoring mechanisms were engaged (Botvinick et al., 2001; Ridderinkhof et al., 2004). Thus, controlled Flanker paradigms using domain-specific, sports-related stimuli may provide a useful approximation for investigating interference control in sport contexts.

Developmental factors provide a likely explanation for the limited group effects. Adolescence is characterized by ongoing maturation of the prefrontal cortex and frontoparietal control networks that underpin inhibition (Arain et al., 2013, Casey et al., 2005, Tervo-Clemmens et al., 2023). Sports-related neural adaptations may therefore be latent or emerging, becoming more evident only in adulthood when neural specialization stabilizes (Gordon-Murer et al., 2021, Holfelder et al., 2020). Furthermore, individual variability in adolescent development which is likely linked to hormonal, educational, and environmental factors, can overshadow subtle sports-specific cognitive differences (Gerván et al., 2024, Sammy Ahmed et al., 2024). Longitudinal research is thus essential to disentangle maturational from training-related effects.

The main effect of the stimulus shows a difference between soccer and the abstract and handball stimuli. Classical flanker-task models assume participants direct attention first to the central target stimulus (e.g., letter or arrow) and ignore flankers (Eriksen & Eriksen, 1974). In our sports-specific stimulus set, however, the salient feature may not simply lie at the geometric centre (e.g., head) but may shift toward motor-relevant zones (e.g., feet for soccer players). Accordingly, it is possible that attention in the football-foot condition was directed initially toward foot position rather than head position, thereby altering the standard locus of processing and interference dynamics. Although we lack direct eye-tracking data to confirm this attentional shift, the strong main effect of stimulus and interaction trends suggests that such effector- and domain-driven attention allocation may play a role.

The strong main effect of response on reaction times indicates that performance varied substantially across effectors, with slower reaction times for hand and foot responses compared to finger responses. Such differences are consistent with well-established findings that motor execution speed depends on both the size and complexity of the effector system involved (Burle et al., 2004, Schmidt & Lee, 2020). In flanker and choice-reaction paradigms, distal effectors such as fingers typically yield faster responses due to lower inertial load and shorter corticospinal conduction distances (Anson, 1989, Pfister et al., 2014). In contrast, foot responses involve larger muscles and longer transmission times, leading to systematically increased latencies (Simonen et al., 1995). Beyond biomechanics, effector differences may also reflect variations in cortical representation and sensorimotor precision: finger movements engage highly specialized motor areas with dense cortico-cortical connectivity, whereas foot actions rely on less differentiated control regions (Schieber, 2001).

In the context of the present study, these response-dependent differences suggest that interference control is tightly coupled with motor-execution constraints. The finding that soccer players showed somewhat larger distinctions between finger and hand responses than the other groups could indicate that habitual foot-dominant training modifies cross-effector coordination or attentional allocation during upper-limb responses (Heppe & Zentgraf, 2019, Swinnen & Wenderoth, 2004). More broadly, the robust response effect underscores the need to consider motor-specific factors in executive function research, as effector selection itself alters the temporal dynamics of inhibition and response selection (Gálvez-García et al., 2018, Tecilla et al., 2022).

A direct comparison with Heppe & Zentgraf (2019) helps contextualize the absence of clear group effects in our data. Their study used the Stop-Signal Task, which measures action cancellation, whereas our Flanker task taps interference control, meaning the two paradigms target partly different inhibitory mechanisms. They also tested fully mature adult athletes, while our 15–18-year-old participants are still in a developmental phase in which inhibitory functions continue to mature, potentially reducing observable sport-related advantages. Developmental work on stop-signal performance suggests that response inhibition (e.g., interference control) refines into early adulthood, which may contribute to the divergent findings. Moreover, the adult athletes in Heppe & Zentgraf’s (2019) study competed at a higher and more homogeneous expertise level than our youth athletes. These methodological, developmental, and expertise-related differences likely explain why their study revealed clear group effects while ours did not.

The age-adjusted reanalysis further supports this interpretation. Because the groups differed significantly in age, and because adolescence is a developmental period in which executive functions are still maturing, age was included as a covariate in additional analyses. Importantly, controlling for age did not change the central pattern of findings. For response times, age significantly contributed to performance, indicating that developmental differences were related to processing speed. However, the critical group-related interactions relevant to the transfer hypothesis remained non-significant after age adjustment, including the group × congruency × stimulus, group × congruency × response, and group × congruency × stimulus × response interactions. Similarly, for accuracy, age was not a significant covariate, and the critical group-related interactions remained non-significant. At the same time, the robust task-related effects of congruency, stimulus type, and response modality remained significant for both response times and accuracy. Thus, the absence of consistent sport-specific transfer effects cannot be explained simply by age differences between groups. Rather, the age-adjusted findings strengthen the conclusion that performance in the present paradigm was primarily shaped by task-specific perceptual and motor demands, whereas group membership played only a minor role.

The significant congruency × stimulus interaction reflects the observation that the congruence effect varies in magnitude depending on the stimulus. Although the classic flanker effect (incongruent > congruent) was present for all stimulus types, the interference was attenuated for the sport-specific conditions, indicating that ecological manipulations altered the nature of interference processing. The slower overall responses to handball and soccer figures suggest that ecologically valid stimuli impose greater perceptual and cognitive demands, likely due to increased visual complexity and the activation of action-related representations (Davelaar, 2012, Kalén et al., 2021, Musculus et al., 2022). In line with the embodied cognition framework (Barsalou, 2008) and models of perception–action coupling in sport (Mann et al., 2007, Hüttermann et al., 2014), sport-specific figures may engage broader neural networks encompassing visuospatial, attentional, and motor areas, thereby increasing processing time while simultaneously narrowing interference effects. By contrast, abstract arrow stimuli are perceptually simple and highly overlearned, eliciting faster but less contextually integrated responses (Ridderinkhof et al., 2021). Together, these findings suggest that increasing ecological validity enhances processing demands yet refines selective attention, leading to a reduced flanker effect despite slower overall reaction times.

The findings underscore two key implications. First, ecological validity is important: interference control performance is highly context-dependent and varies substantially with perceptual and motor task demands. Traditional paradigms using simple, abstract stimuli likely underestimate real-world cognitive–motor integration. Second, the lack of robust group differences suggests caution in using executive function tasks for talent identification or selection in youth sports (Fransen, 2024, Reinhard et al., 2025). Rather than relying on domain-general cognitive measures, training programs should emphasize context-specific, perceptually grounded exercises that directly engage the sensory–motor couplings central to sports performance (Heilmann & Schubert, 2025).

Next, we would like to acknowledge the limitations of our study. The cross-sectional design does not permit causal conclusions regarding training-related adaptations. Differences, or the lack thereof, could reflect pre-existing individual differences rather than consequences of sports participation. A longitudinal design following athletes across training years would be necessary to establish causality. The adolescent sample represents another limitation. Adult athletes show more pronounced adaptations that were not yet evident in our younger participants. Furthermore, accuracy rates were generally high, raising the possibility of ceiling effects that may have masked potential group differences.

A further limitation concerns the static nature of the stimulus material. Although the use of photographs increased domain specificity compared to traditional abstract stimuli, real sport environments are characterized by dynamic and continuously evolving action sequences. Dynamic information provides kinematic cues that are critical for anticipation and action selection and may engage partially different perceptual and visuomotor processing mechanisms than static displays. Consequently, the present paradigm captures only a controlled approximation of sport-specific perception–action demands. Future research should therefore extend interference-control paradigms toward video-based or interactive designs to better approximate the temporal dynamics of real competitive situations and to examine whether expertise effects emerge more clearly under dynamic conditions.

Furthermore, handedness was not formally assessed or controlled for, which may have influenced performance differences between manual response conditions and should be addressed in future research.

Despite these limitations, the study provides important contributions to the literature. It demonstrates convincingly that interference control performance is sensitive to ecological manipulations of task condition, reinforcing the argument that executive functions are not fixed capacities but context-dependent processes. At the same time, it tempers claims of broad sports-related cognitive benefits, at least in adolescent populations. This dual message is significant for both theoretical debates in cognitive neuroscience and practical considerations in sports science.

In conclusion, the present study demonstrates that interference-control performance in adolescents is strongly influenced by stimulus characteristics and response effectors, highlighting the importance of perceptual–motor task demands in executive-function research. Despite these robust task-related effects, soccer and handball players did not show consistent performance advantages over controls, suggesting that sport-related cognitive adaptations may be subtle, context-dependent, or not yet fully developed during adolescence. By specifying the conditions under which sport-specific effects may emerge, the study contributes to a more differentiated understanding of how athletic training interacts with cognitive development. Future research should employ longitudinal approaches and more ecologically representative paradigms, and may benefit from combining behavioral and neurophysiological measures to better disentangle the roles of training, maturation, and neural plasticity in shaping executive functions across development.

## Data Availability

The data that support the findings of this study are openly available at https://osf.io/c3h67/overview? view_only=635a285a86e1486eae8435d9baea92b8.

## References

[CR1] Abernethy, B., Gill, D. P., Parks, S. L., & Packer, S. T. (2001). Expertise and the perception of kinematic and situational probability information. *Perception,**30*(2), 233–252. 10.1068/p2872 11296504 10.1068/p2872

[CR2] Albaladejo-García, C., García-Aguilar, F., & Moreno, F. J. (2023). The role of inhibitory control in sport performance: Systematic review and meta-analysis in stop-signal paradigm. *Neuroscience and Biobehavioral Reviews,**147*, Article 105108. 10.1016/j.neubiorev.2023.105108 36828162 10.1016/j.neubiorev.2023.105108

[CR3] Anson, J. G. (1989). Effects of moment of inertia on simple reaction time. *Journal of Motor Behavior,**21*(1), 60–71. 10.1080/00222895.1989.10735465 15117673 10.1080/00222895.1989.10735465

[CR4] Arain, M., Haque, M., Johal, L., Mathur, P., Nel, W., Rais, A., Sandhu, R., & Sharma, S. (2013). Maturation of the adolescent brain. *Neuropsychiatric Disease and Treatment,**9*, 449–461. 10.2147/NDT.S39776 23579318 10.2147/NDT.S39776 PMC3621648

[CR5] Barsalou, L. W. (2008). Grounded cognition. *Annual Review of Psychology,**59*, 617–645. 10.1146/annurev.psych.59.103006.093639 17705682 10.1146/annurev.psych.59.103006.093639

[CR6] Botvinick, M. M., Braver, T. S., Barch, D. M., Carter, C. S., & Cohen, J. D (2001). Conflict monitoring and cognitive control. *Psychological Review, 108*(3), 624–652. 10.1037/0033-295X.108.3.624 10.1037/0033-295x.108.3.624 11488380

[CR7] Büchel, D., Gokeler, A., Heuvelmans, P., & Baumeister, J. (2022). Increased cognitive demands affect agility performance in female athletes - Implications for testing and training of agility in team ball sports. *Perceptual and Motor Skills,**129*(4), 1074–1088. 10.1177/00315125221108698 35703458 10.1177/00315125221108698 PMC9301166

[CR8] Burle, B., Vidal, F., Tandonnet, C., & Hasbroucq, T. (2004). Physiological evidence for response inhibition in choice reaction time tasks. *Brain and Cognition,**56*(2), 153–164. 10.1016/j.bandc.2004.06.004 15518932 10.1016/j.bandc.2004.06.004

[CR9] Buschman, T. J., & Kastner, S. (2015). From behavior to neural dynamics: An integrated theory of attention. *Neuron,**88*(1), 127–144. 10.1016/j.neuron.2015.09.017 26447577 10.1016/j.neuron.2015.09.017 PMC4604109

[CR10] Cao, L. Z., He, H., Miao, X., & Chi, L. (2024). The contributions of executive functions to decision-making in sport. *International Journal of Sport and Exercise Psychology*, 1–20. 10.1080/1612197X.2024.2371483

[CR11] Casey, B. J., Tottenham, N., Liston, C., & Durston, S. (2005). Imaging the developing brain: What have we learned about cognitive development? *Trends in Cognitive Sciences,**9*(3), 104–110. 10.1016/j.tics.2005.01.011 15737818 10.1016/j.tics.2005.01.011

[CR12] Chanceaux, M., Mathôt, S., & Grainger, J. (2014). Effects of number, complexity, and familiarity of flankers on crowded letter identification. *Journal of Vision*. 10.1167/14.6.7 25384390 10.1167/14.6.7

[CR13] Davelaar, E. J. (2012). When the ignored gets bound: Sequential effects in the flanker task. *Frontiers in Psychology*, *3*, 552. 10.3389/fpsyg.2012.00552 23293616 10.3389/fpsyg.2012.00552 PMC3534361

[CR14] Diamond, A. (2013). Executive functions. *Annual Review of Psychology,**64*, 135–168. 10.1146/annurev-psych-113011-143750 23020641 10.1146/annurev-psych-113011-143750 PMC4084861

[CR15] Eriksen, B. A., & Eriksen, C. W. (1974). Effects of noise letters upon the identification of a target letter in a nonsearch task. *Perception & Psychophysics*, *16*(1), 143–149. 10.3758/BF03203267

[CR16] Fransen, J. (2024). There is no supporting evidence for a far transfer of general perceptual or cognitive training to sports performance. *Sports Medicine (Auckland, N.Z.),**54*(11), 2717–2724. 10.1007/s40279-024-02060-x 38907178 10.1007/s40279-024-02060-x PMC11560981

[CR17] Gálvez-García, G., Albayay, J., Rehbein, L., Bascour-Sandoval, C., & Michael, G. A. (2018). Response inhibition as a function of movement complexity and movement type selection. *Frontiers in Psychology,**9*, Article 2290. 10.3389/fpsyg.2018.02290 30534099 10.3389/fpsyg.2018.02290 PMC6275418

[CR18] Gerván, P., Oláh, G., Utczás, K., Tróznai, Z., Berencsi, A., Gombos, F., & Kovács, I. (2024). The influence of relative pubertal maturity on executive function development in adolescent girls. *Scientific Reports*, *14*(1), 28140. 10.1038/s41598-024-71768-7 39548095 10.1038/s41598-024-71768-7 PMC11568130

[CR19] Gordon-Murer, C., Stöckel, T., Sera, M., & Hughes, C. M. L. (2021). Developmental Differences in the Relationships Between Sensorimotor and Executive Functions. *Frontiers in Human Neuroscience*, *15*, 714828. 10.3389/fnhum.2021.714828 34456700 10.3389/fnhum.2021.714828 PMC8387672

[CR20] Güldenpenning, I., Kunde, W., & Weigelt, M. (2017). How to Trick Your Opponent: A Review Article on Deceptive Actions in Interactive Sports. *Frontiers in Psychology*, *8*, 917. 10.3389/fpsyg.2017.00917 28620336 10.3389/fpsyg.2017.00917 PMC5449506

[CR21] Gu, Q., Zou, L., Loprinzi, P. D., Quan, M., & Huang, T. (2019). Effects of Open Versus Closed Skill Exercise on Cognitive Function: A Systematic Review. *Frontiers in Psychology*, *10*, 1707. 10.3389/fpsyg.2019.01707 31507472 10.3389/fpsyg.2019.01707 PMC6718477

[CR22] Haugan, J. A., Lervold, K., Kaalvik, H., & Moen, F. (2025). A scoping review of empirical research on executive functions and game intelligence in soccer. *Frontiers in Psychology*, *16*, 1536174. 10.3389/fpsyg.2025.1536174 40230991 10.3389/fpsyg.2025.1536174 PMC11994698

[CR23] Heilmann, F., & Schubert, T. (2025). The influence of specific cognitive training in virtual reality on the inhibition of elite young ice hockey players. *Frontiers in Sports and Active Living*. 10.3389/fspor.2025.1682165 41245651 10.3389/fspor.2025.1682165 PMC12611849

[CR24] Heilmann, F., Weinberg, H., & Wollny, R. (2022). The impact of practicing open- vs. closed-skill sports on executive functions-A meta-analytic and systematic review with a focus on characteristics of sports. *Brain Sciences*. 10.3390/brainsci12081071 36009134 10.3390/brainsci12081071 PMC9406193

[CR25] Heppe, H., & Zentgraf, K. (2019). Team handball experts outperform recreational athletes in hand and foot response inhibition: A behavioral study. *Frontiers in Psychology,**10*, Article 971. 10.3389/fpsyg.2019.00971 31133925 10.3389/fpsyg.2019.00971 PMC6524689

[CR26] Holfelder, B., Klotzbier, T. J., Eisele, M., & Schott, N. (2020). Hot and Cool Executive Function in Elite- and Amateur- Adolescent Athletes From Open and Closed Skills Sports. *Frontiers in Psychology*, *11*, 694. 10.3389/fpsyg.2020.00694 32373029 10.3389/fpsyg.2020.00694 PMC7177013

[CR27] Hülsdünker, T., Friebe, D., Giesche, F., Vogt, L., Pfab, F., Haser, C., & Banzer, W. (2023). Validity of the SKILLCOURT^®^ technology for agility and cognitive performance assessment in healthy active adults. *Journal of Exercise Science & Fitness,**21*(3), 260–267. 10.1016/j.jesf.2023.04.003 37497363 10.1016/j.jesf.2023.04.003 PMC10366450

[CR28] Hüttermann, S., Memmert, D., & Bock, A. (2014). Individual differences in attentional capability are linked to creative decision making. *Journal of Applied Research in Memory and Cognition, 3*(2), 101–109. 10.1016/j.jarmac.2014.03.003

[CR29] Kalén, A., Bisagno, E., Musculus, L., Raab, M., Pérez-Ferreirós, A., Williams, A. M., Araújo, D., Lindwall, M., & Ivarsson, A. (2021). The role of domain-specific and domain-general cognitive functions and skills in sports performance: A meta-analysis. *Psychological Bulletin,**147*(12), 1290–1308. 10.1037/bul0000355 35404636 10.1037/bul0000355

[CR30] Kibele, A. (2006). Non-consciously controlled decision making for fast motor reactions in sports—A priming approach for motor responses to non-consciously perceived movement features. *Psychology of Sport and Exercise,**7*(6), 591–610. 10.1016/j.psychsport.2006.05.001

[CR31] Mann, D. T. Y., Williams, A. M., Ward, P., & Janelle, C. M. (2007). Perceptual-cognitive expertise in sport: A meta-analysis. *Journal of Sport & Exercise Psychology,**29*(4), 457–478. 10.1123/jsep.29.4.457 17968048 10.1123/jsep.29.4.457

[CR32] Musculus, L., Lautenbach, F., Knöbel, S., Reinhard, M. L., Weigel, P., Gatzmaga, N., Borchert, A., & Pelka, M. (2022). An assist for cognitive diagnostics in soccer: Two valid tasks measuring inhibition and cognitive flexibility in a soccer-specific setting with a soccer-specific motor response. *Frontiers in Psychology,**13*, Article 867849. 10.3389/fpsyg.2022.867849 35432102 10.3389/fpsyg.2022.867849 PMC9009540

[CR33] Pfister, M., Lue, J. C. L., Stefanini, F. R., Falabella, P., Dustin, L., Koss, M. J., & Humayun, M. S. (2014). Comparison of reaction response time between hand and foot controlled devices in simulated microsurgical testing. *BioMed Research International*. 10.1155/2014/769296 25136619 10.1155/2014/769296 PMC4109600

[CR34] Reinhard, M. L., Martin, L., Mann, D. L., & Höner, O. (2025). The role of generic cognitive skills: An empirical investigation into the association between generic and sport-specific cognitive skills and playing level in youth football. *Journal of Science and Medicine in Sport*, *28*(7), 587–593. 10.1016/j.jsams.2025.01.010 10.1016/j.jsams.2025.01.010 40044540

[CR35] Ridderinkhof, K. R., van den Wildenberg, M. W., Segalowitz, C., & Carter, S. G. (2004). Neurocognitive mechanisms of cognitive control: The role of prefrontal cortex in action selection, response inhibition, performance monitoring, and reward-based learning. *Brain and Cognition, 56*(2), 129–140. 10.1016/j.bandc.2004.09.016 10.1016/j.bandc.2004.09.016 15518930

[CR36] Ridderinkhof, K. R., Wylie, S. A., van den Wildenberg, W. P. M., Bashore, T. R., & van der Molen, M. W. (2021). The arrow of time: Advancing insights into action control from the arrow version of the Eriksen flanker task. *Attention, Perception & Psychophysics,**83*(2), 700–721. 10.3758/s13414-020-02167-z 10.3758/s13414-020-02167-z PMC7884358 33099719

[CR37] Sammy Ahmed, D., Kelly, N., Waters, & Natasha Chaku. (2024). *Executive Functioning*10.13140/RG.2.2.26371.11041.

[CR38] Scharfen, H. E., Lehmann, T., Büchel, D., & Baumeister, J. (2022). Cortical responses to sport-specific stimuli in a standing stop signal task. *Psychology of Sport and Exercise,**63*, Article 102250. 10.1016/j.psychsport.2022.102250

[CR39] Schieber, M. H. (2001). Constraints on somatotopic organization in the primary motor cortex. *Journal of Neurophysiology*, *86*(5), 2125–2143. 10.1152/jn.2001.86.5.2125 11698506 10.1152/jn.2001.86.5.2125

[CR40] Schmidt, R. A., & Lee, T. D. (2020). *Motor learning and performance: From principles to application* (Sixth edition). Human Kinetics.

[CR41] Simonen, R. L., Videman, T., Battié, M. C., & Gibbons, L. E. (1995). Comparison of foot and hand reaction times among men: A methodologic study using simple and multiple-choice repeated measurements. *Perceptual and Motor Skills,**80*(3 Pt 2), 1243–1249. 10.2466/pms.1995.80.3c.1243 7478883 10.2466/pms.1995.80.3c.1243

[CR42] Simonet, M., Ruggeri, P., & Barral, J. (2020). Effector-specific characterization of brain dynamics in manual vs. oculomotor Go/NoGo tasks. *Frontiers in Human Neuroscience,**14*, Article 600667. 10.3389/fnhum.2020.600667 33343320 10.3389/fnhum.2020.600667 PMC7744377

[CR43] Simonet, M., Beltrami, D., & Barral, J. (2023). Inhibitory control expertise through sports practice: A scoping review. *Journal of Sports Sciences,**41*(7), 616–630. 10.1080/02640414.2023.2230713 37409697 10.1080/02640414.2023.2230713

[CR44] Swinnen, S. P., & Wenderoth, N. (2004). Two hands, one brain: Cognitive neuroscience of bimanual skill. *Trends in Cognitive Sciences,**8*(1), 18–25. 10.1016/j.tics.2003.10.017 14697399 10.1016/j.tics.2003.10.017

[CR45] Tecilla, M., Guerra, A., Rocchi, L., Määttä, S., Bologna, M., Herrojo Ruiz, M., Biundo, R., Antonini, A., & Ferreri, F. (2022). Action selection and motor decision making: Insights from transcranial magnetic stimulation. *Brain Sciences*. 10.3390/brainsci12050639 35625025 10.3390/brainsci12050639 PMC9139261

[CR46] Tervo-Clemmens, B., Calabro, F. J., Parr, A. C., Fedor, J., Foran, W., & Luna, B. (2023). A canonical trajectory of executive function maturation from adolescence to adulthood. *Nature Communications,**14*(1), 6922. 10.1038/s41467-023-42540-8 37903830 10.1038/s41467-023-42540-8 PMC10616171

[CR47] Voss, M. W., Kramer, C. R., Basak, J., Prakash, SY., Roberts, W. C., Kramer, A. F. (2010). Are expert athletes ‘expert’ in the cognitive laboratory? A meta-analytic review of cognition and sport expertise. *Applied Cognitive Psychology, 24*(6), 812–826. 10.1002/acp.1588

[CR48] Weigelt, M., Güldenpenning, I., & Steggemann-Weinrich, Y. (2020). The Head-Fake Effect in Basketball Is Based on the Processing of Head Orientation, but Not on Gaze Direction. *Psychology, 11*(10), 1493–1510. 10.4236/psych.2020.1110095

[CR49] Wilcox, R. R. (2011). *Introduction to robust estimation and hypothesis testing*. Academic.

[CR50] Xiong, Q., & Song, D. L. (2024). Neuromechanical proficiency in elite performance decision-making: An event-related potential (ERP) analysis. *SLAS Technology,**29*(5), Article 100171. 10.1016/j.slast.2024.100171 39067818 10.1016/j.slast.2024.100171

